# Clinical and radiographic outcomes of the treatment of adolescent idiopathic scoliosis with segmental pedicle screws and combined local autograft and allograft bone for spinal fusion: a retrospective case series

**DOI:** 10.1186/1471-2474-11-159

**Published:** 2010-07-14

**Authors:** Xiaoming Yang, Hongguang Xu, Ming Li, Suxi Gu, Xiutong Fang, Jingjie Wang, Jianqiang Ni, Dajiang Wu

**Affiliations:** 1 Department of Orthopedics, Changhai Hospital, Second Military Medical University, No. 168 Shanghai Road, Shanghai, PRC. 200433; 2 Department of Orthopedics, Yijishan Hospital, Wannan Medical College, No. 92 Zheshan West Road, Wuhu, Anhui province, PRC. 241001

## Abstract

**Background:**

High morbidity has been reported with iliac crest bone graft harvesting; however, donor bone is typically necessary for posterior spinal fusion. Autograft bone combined with allograft may reduce the morbidity associated with iliac crest bone harvesting and improve the fusion rate. Our aim in this study was to determine the presence of complications, pseudarthrosis, non-union, and infection using combined *in situ *local autograft bone and freeze-dried cancellous allograft bone in patients undergoing posterior spinal fusion for the treatment of adolescent idiopathic scoliosis.

**Methods:**

A combination of *in situ *local autograft bone and freeze-dried cancellous allograft blocks were used in 50 consecutive patients with adolescent idiopathic scoliosis treated by posterior fusion and Moss Miami pedicle screw instrumentation. Results were assessed clinically and radiographically and quality of life and functional outcome was evaluated by administration of a Chinese version of the SRS-22 survey.

**Results:**

There were 41 female and 9 male patients included for analysis with an average age of 14.7 years (range, 12-17). All patients had a minimum follow-up of 18 months (range, 18 to 40 months). The average preoperative Cobb angle was 49.8° (range, 40° to 86°). The average number of levels fused was 9.8 (range, 6-15). Patients had a minimum postoperative follow-up of 18 months. At final follow-up, the average Cobb angle correction was 77.8% (range, 43.4 to 92.5%). There was no obvious loss in the correction, and the average loss of correction was 1.1° (range, 0° to 4°). There was no pseudarthrosis and no major complications.

**Conclusions:**

*In situ *autograft bone combined with allograft bone may be a promising method enhances spinal fusion in AIS treated with pedicle screw placement. By eliminating the need for iliac crest bone harvesting, significant morbidity may be avoided.

## Background

The iliac crest is considered the best source of autograft bone for procedures treating bone nonunion, spinal fusion, and specifically posterior spinal fusion in corrective surgery for adolescent idiopathic scoliosis (AIS). However, studies have reported numerous complications associated with harvesting iliac crest bone including bleeding, infection, gait disturbance, neurological injury, fracture, and persistent donor site pain, with an incidence ranging from 24% to 29% [1-3]. The high morbidity of iliac crest bone graft harvesting has limited its application. Allograft bone has the advantages of adequate supply and variety of type, and reports indicate that allograft bone is a suitable alternative to autogenous bone grafting for AIS corrective surgical procedures [4,5].

Our aim in this study was to determine the presence of complications, pseudarthrosis, non-union, and infection using combined in situ local autograft bone and freeze-dried cancellous allograft bone in patients undergoing posterior spinal fusion for the treatment of AIS with segmental pedicle screws and dual rod instrumentation.

## Methods

### Subjects

In this retrospective study, the records of 50 consecutive patients with AIS who underwent posterior spinal fusion and MOSS^® ^MIAMI pedicle screw instrumentation (DePuy Spine, Raynham, MA, US) at our hospital from November 2004 to June 2006 were reviewed. Criteria for surgical correction of AIS included: 1) Conservative treatment ineffective at controlling symptoms, 2) Patient not satisfied with appearance, 3) Cobb angle >40°, 4) Risser score ≥3°, and 5) No history of spinal surgery. This retrospective review of medical records was approved by the Institutional Review Board of our hospital.

### Surgical techniques

All operations were performed by the same surgeon (ML). Anterior discectomy and release was performed in patients with rigid major curves >75° with a correction <50% in bending radiograms. A standard posterior midline surgical incision was made and the spine exposed using a combination of blunt subperiosteal dissection and electrocautery. After removal of all soft tissue, autograft bone was obtained from the spinous processes, laminae, and transverse processes of all vertebrae which did not support instrumentation. Pedicle screws were inserted with the free hand pedicle screw placement technique as described by Kim et al. [6]. Once the screws were in place, intraosseous placement was confirmed via a C-arm image intensifier.

With concave rod insertion, curve correction was achieved with rod rotation, *in situ *translational correction, compression and/or distraction, and direct apical vertebral body derotation, which were used to provide 3-dimensional correction of the deformity. The lamina corticalis of all laminae and articular processes in the fusion range were raised by osteotomy.

Allograft bone was purchased from Beijing Xinkangcheng Medicine Development Center (Beijing, P.R. China). Just prior to beginning the procedure of bone planting, allograft bones were soaked with 0.9% physiological saline for several minutes. Local autograft bone was cut into match-like sticks, combined with allograft bone, and carefully packed onto the prepared surfaces. The purpose of the allograft bone was to be a scaffold for bone growth. The amount of allograft was determined by the length of the fusion segment such that the allograft combined with autograft completely covered the bone bed. A drainage tube was placed and the wound sutured in layers. Patients were encouraged to stand up and walk by the fourth or fifth postoperative day. No external brace was used after surgery.

### Assessment of outcome

Erect anteroposterior and lateral radiographs of the whole spinal column were taken preoperatively, at 3, 6, and 12 months postoperatively, and at every 6-12 month follow-up visit thereafter. Possible pseudarthrosis was determined by 1) persistent midline moderate-to-severe back pain, 2) a defect in the fusion mass or an unfused facet visible on radiograph, and 3) curve progression >10° from the initial erect postoperative radiograph [7]. Quality of life and functional outcome was evaluated by administration of a Chinese version of the SRS-22 survey [8] at the last follow-up visit.

## Results

There were 41 female and 9 male patients with an average age of 14.7 years (range, 12-17) included in the analysis. The numbers of patients with Lenke type 1, 2, 3, 5, and 6 curves were 20, 3, 9, 14, and 4, respectively. The average preoperative Cobb angle was 49.8° (range, 40° to 86°). The average number of levels fused was 9.8 (range, 6-15). The average Cobb angle correction was 77.8% (range, 43.4% to 92.5%). The results of postoperative and follow-up Cobb angle are presented in Table 1.

**Table 1:** Pre- and postoperative radiographic measurements of the 50 patients

	**Proximal Curve**^**a **^***n *= 37**	**Distal Curve**^**b **^***n *= 31**	Fusion levels N = 50
Postoperative correction rate (%)	71.7 ± 15.7	78.9 ± 11.5	N/A
Cobb angle at last follow-up (°)	15.1 ± 10.8	11.5 ± 7.8	N/A
Correction rate at last follow-up (%)	70.0 ± 16.4	77.0 ± 12.2	N/A

Data presented as mean ± standard deviation (SD).

N/A, not available.

^a ^Including thoracic curve and proximal thoracic curve.

^b ^Including distal thoracic, thoracic/lumbar, and lumbar curve.

**Figure 1:**
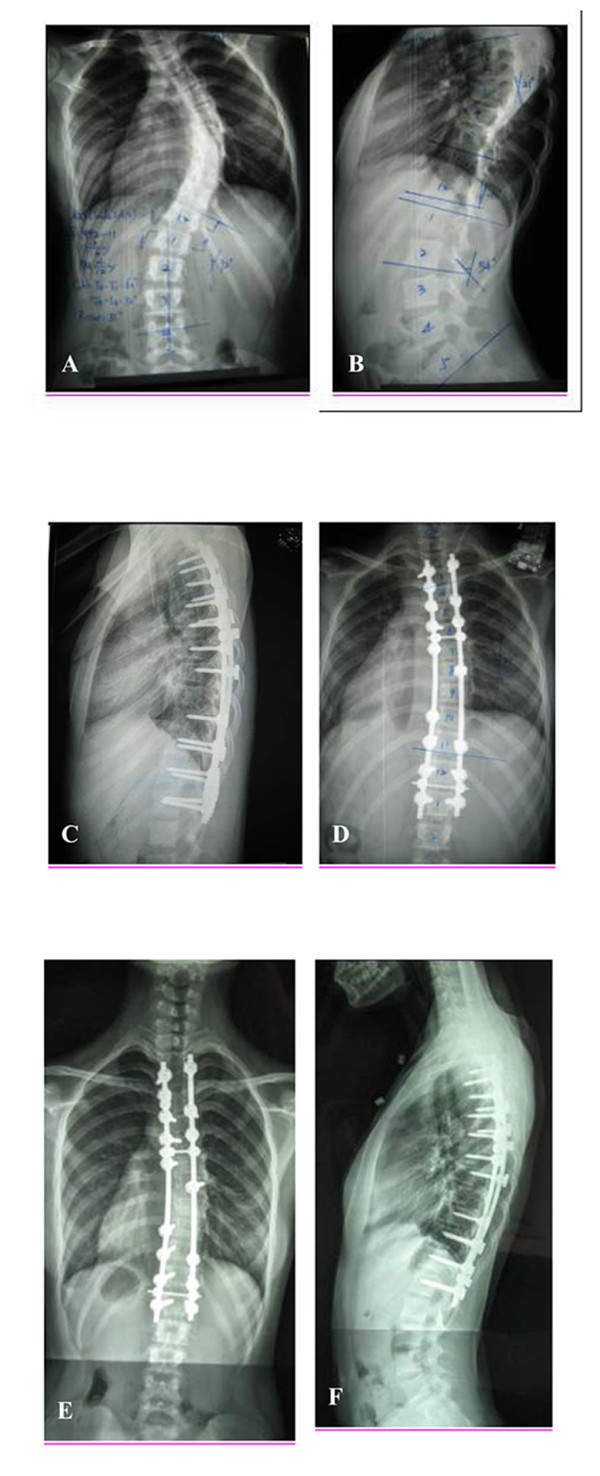
**Patient is a 13-year-old girl with Lenke 1AN adolescent idiopathic scoliosis**. A, B: Preoperatively, a right thoracic curve of 60° from T4 to T11 is noted. C, D: Two weeks postoperatively, the thoracic curve is corrected to 21° and thoracic kyphosis is well maintained. E, F: At 2 years postoperatively, there is no correction loss in the frontal and lateral planes, all treated levels are well-fused, and no cracks are noted.

All patients had a minimum follow-up of 18 months (range, 18 to 40 months). At the last follow-up, fusion was found to be complete in all patients, and no cracks were noted. No Cobb angle change >10° between an immediate postoperative radiograph and the last follow-up erect radiograph was found. There was no obvious loss in the correction, and the average loss of correction was 1.1° (range, 0° to 4°). No pull-out of pedicle screws or broken rods was discovered during the follow-up period. No revision surgeries were required and no pseudarthrosis was found during the follow-up period. No neurologic, cardiac, pulmonary, or infectious complications occurred. There were no cases of infection or other adverse consequences due to excessive exudate. Radiographs of a representative case are presented in the Figure 1. The results of the SRS-22 scores at the last follow-up are presented in Table 2. No patients had complaints of back pain and all returned to normal school study a month after surgery, and had more confidence in daily life.

**Table 2:** SRS-22 scores at last follow-up

Domain	Average Score	Range
Pain	4.32	3.8-5
Mental health	4.37	3.8-5
Function/activity	4.56	4.0-5
Self-image/appearance	4.48	3.8-5
Satisfaction of management	3.80	2.5-5

## Discussion

Iliac crest bone graft (ICBG) is effective in the augmentation of bone healing in spinal fusion procedures; however, a high complication rate harvesting iliac crest bone and small available quantities restrict the use of ICBG. *In situ *local autograft bone harvesting avoids the morbidity of ICBG, and also has been shown to have osteoinductive activity. Violas et al. [9] reported the results of 25 AIS cases treated by Cotrel-Dubousset instrumentation using only local autograft bone. With a mean follow-up of 6 years, all patients did well and no loss of correction or pseudarthrosis was noted. Blanco et al. [4] reported the results of 25 AIS patients who underwent posterior spine fusion with freeze-dried allograft bone and Cotrel-Dubousset instrumentation. With the minimum follow up of 3 years, no loss of correction or pseudarthrosis was identified. Studies have also shown allograft bone to be as effective as autograft bone in posterior spinal fusion [5,10]. In our study, we found the combination of autograft and allograft bone provided good results in the treatment of AIS, i.e., no failure of fusion were seen on radiographs, percent correction was well maintained, and no patients complained of back pain.

Allograft bone is available in a number of shapes, sizes, and types, including fresh, fresh-frozen, or freeze-dried cancellous or cortical bone [11]. *In situ *local autograft bone with allograft not only maintains the advantages of autograft, but also increases the supply of graft bone. Other potential benefits include decreased operative time, blood loss, and donor site morbidity as well as cosmetic advantages.

While the availability of allograft bone compensates for the lack of autologous bone, there is concern regarding the risk of bacterial contamination and viral transmission. Asselmeier et al. [12] reviewed more than 1,000,000 freeze-dried allograft transplantations performed since 1951 and found no documented cases of HIV or other viral transmission. Studies have indicated the risk of disease transmission is greater with fresh and fresh-frozen allograft bone than with freeze-dried bone [11,13]. It is because of this data that we choose to use freeze-dried bone in our patients. Though the follow-up period is relatively short, we have not found any evidence of infection caused by the allograft bone in our patients.

In 1995, Suk et al. [14] first reported the use of all pedicle screws in the treatment of AIS. They found significantly better coronal, sagittal, and hypokyphosis correction for all-screw constructs versus all-hook instrumentation. Similar results were reported by other authors [15]. Lowenstein et al. [16] also compared the result between all-screw and hybrid thoracic hook lumbar screw constructs in treatment of 34 patients with AIS. A trend was observed toward better correction of the main thoracic curve in the all-screw group and the all-screw group demonstrated a significant decrease in kyphosis. Compared to hooks, pedicle screws offer 3-column purchase and a longer moment arm. Instrumentation of each vertebral level with pedicle screws on the correcting side allows a more rigid fixation and reduces the stress on any one particular screw during manipulation. This method also allows control of each instrumented segment, including the apex of the curve, which allows selective intersegmental compression, distraction, translation, and rotation [17].

While segmental pedicle screw fixation can achieve good stability, failure can occur if adequate inner fixation does not occur. The application of allograft bone can contribute to good integration and fusion of the fixed segments; however, few studies have examined the use of allograft bone in posterior spinal fusion when pedicle screws are used. Betz et al. [18] compared the results of allograft versus no bone graft with a posterior hook system for the treatment of idiopathic scoliosis in patients with an average age of 14.5 years and an average preoperative curve of 52.6°. At 2-year follow-up, there was no difference between the groups with regards to pseudoarthosis rate and loss of correction. The overall average correction was 63% and overall pseudoarthosis rate was 1.3%, results comparable to that of the current study.

Lower pseudarthrosis rates have been reported since the development of segmental fixation [9]. Lenke et al. [19] reported on 76 AIS patients who underwent posterior spine fusion with Cotrel-Dubousset instrumentation. With an average of 6 years follow-up, no pseudarthrosis was identified. Because a stronger correction force is provided by more rigid fixation, this technique provides better fusion. In contrast to hooks, pedicle screws offer more rigid correction. In short, screws provide a more stable environment, which is good for fusion.

Of note, is that none of the patients had significant complaints of back pain. Adult patients with scoliosis often complain of back pain during follow-up. We believe this may be because adolescents are more concerned with the correction of their body shape and appearance and are thus more tolerant of pain.

There are limitations to the study that must be considered. First, the average follow-up of 18 months is relatively short and the number of patients is small. Thought the results are very encouraging, follow-up of 3-5 years with a larger cohort is required to adequately evaluate outcome. Second, most of the patients did not have severe scoliosis, i.e., the average Cobb angle was 49.8°. The results may not be reproducible with more severe disease. Also, all procedures were performed by the same surgeon. Though variation in outcome was minimized by this, different surgeons might have varied outcomes.

## Conclusions

In summary, this retrospective case-series indicates that *in situ *autograft bone combined with allograft bone may be a promising method to enhance spinal fusion in AIS treated with pedicle screw placement. By eliminating the need for iliac crest bone harvesting, significant morbidity may be avoided. Further prospective, randomized, case controlled studies using systematic inclusion criteria and follow-up are needed to determine the usefulness of the procedure.

## Competing interests

The authors declare that they have no competing interests.

## Authors' contributions

All authors read and approved the final manuscript. ML designed the study. XMY carried out all statistical analysis and drafted the manuscript. JJW and XTF did the work of data collection. JQN and DJW took charge of follow-up data management. SXG made the English grammar correction in manuscript editing.

## Pre-publication history

The pre-publication history for this paper can be accessed here:

http://www.biomedcentral.com/1471-2474/11/159/prepub
